# Heterogeneity of the 5′-end in plant mRNA may be involved in mitochondrial translation

**DOI:** 10.3389/fpls.2013.00517

**Published:** 2013-12-17

**Authors:** Tomohiko Kazama, Yusuke Yagi, Kinya Toriyama, Takahiro Nakamura

**Affiliations:** ^1^Laboratory of Environmental Biotechnology, Graduate School of Agricultural Science, Tohoku UniversitySendai, Japan; ^2^Faculty of Agriculture, Kyushu UniversityFukuoka, Japan

**Keywords:** plants, mitochondria, translation, RNA processing, protein-RNA interaction

## Introduction

Genomic organization and gene expression system of plant mitochondria are distinct from those of other eukaryotes, including animals, even though of chloroplast, another organelle in plants that contain its own genomes. Recent research has revealed the significance of the control of gene expression at the RNA level, including the formation of the 5′- and 3′-ends of mitochondrial mRNA. Although the details of gene expression in mitochondria are not the same as in chloroplasts, huge numbers of nuclear-encoded pentatricopeptide repeat (PPR) proteins are transported in both mitochondria and chloroplasts and control their gene expression. (See other articles in this issue.)

## CMS/*Rf* system

Research on mitochondrial gene expression in plants has focused on understanding cytoplasmic male sterility (CMS), which is caused by the incompatibility between the mitochondrial and nuclear genomes. The mitochondrial genome allows active recombination of genes to occur and easily generates new protein-coding genes. Expression of such a gene sometimes causes male sterility, and consequently the gene is called a CMS-associated gene. Pollen fertility can be restored by suppressing the expression of the CMS-associated gene with a nuclear-encoded *fertility restorer* (*Rf*) gene. The CMS/*Rf* system is useful in agriculture because it enables easy crossbreeding of varieties to produce hybrid seeds. It is also an excellent model in which to study the nuclear control of mitochondrial gene expression. Extensive research has been conducted to identify CMS-associated genes and *Rf* genes.

One organism that has been studied for this purpose is BT-CMS rice. The BT-CMS/*Rf* system contains the mitochondrial CMS gene *orf79* and the nuclear-encoded *Rf1a* gene, which codes for a PPR protein (Kazama and Toriyama, 2003). The *orf79* gene is co-transcribed with its upstream gene, *atp6*. The protein of *Rf1a* promotes the cleavage of *atp6*–*orf79* co-transcribed mRNAs. The cleavage prevents the translation of *orf79* (Kazama et al., 2008).

A similar system exists in a *Brassica* CMS of Ogura. The mitochondrial genome contains the CMS-associated gene *orf138*, which is co-transcribed as *orf138*–*atp8* (Bonhomme et al., 1992). The amount and processing pattern of the co-transcribed mRNA is not affected by the presence or absence of the *Rf* gene, which codes for a PPR protein (Koizuka et al., 2003). The RFo/PPR-B protein is known to be associated with the *orf138* gene containing RNA, suggesting that its function is direct suppression of the translation (Uyttewaal et al., 2008a). In the cases of both rice and the *Brassica* CMS, the translational step seems to be the critical step in the CMS/*Rf* system.

## Translation in plant mitochondria

Hardly anything is known about the translational control of gene expression in plant mitochondria. Regarding the *cis*-regulatory element, the mitochondrial mRNAs do not follow the Shine-Dalgarno sequence that exists in prokaryotic organisms. An early informatics study found three conserved sequence blocks in the 5′ untranslated region (UTR) of mitochondrial RNAs (Pring et al., 1992): block I (GGGAGCAGAG), block II (AGUCUCCCUUUC), and block III [GU (n) CGUUGG]. These blocks generally occur within 100 bases of the 5′ flanking region of the start codons, suggesting that they are involved in mitochondrial translation. However, their functionality has not been evaluated, due to the lack of experimental techniques to study mitochondrial translation.

Recent advances in genetic studies have revealed, although sporadically, the protein factors that are involved in plant mitochondrial translation. The silencing of the nuclear-encoded *rps10* gene, which codes for the mitochondrial ribosomal protein S10, has induced differential translations of mitochondrial transcripts, including over-expression of ribosomal proteins and down-regulation of oxidative phosphorylation subunits (Kwasniak et al., 2013). The PPR protein of the *MPPR6* gene in maize has been shown to interact with the 5′ UTR of the *rps3* mRNA, encoding mitochondrial ribosomal protein S3. This protein may also be involved in 5′ maturation and translational initiation of the *rps3* mRNA. The loss of *MPPR6* results, consequently, in a considerable reduction of mitochondrial translation (Manavski et al., 2012). The loss of translation activity induces general down-regulation of mitochondrial RNA, in contrast with the silencing of the *rps10* gene (Kwasniak et al., 2013). Another PPR protein of the *PPR336* gene has been shown to associate with polysomes in the mitochondria. The mutant plant has unusual polysomal profiles, suggesting that PPR336 could be involved in translation (Uyttewaal et al., 2008b), although the actual mechanism has not been elucidated.

## Putative role of 5′-end heterogeneity of mRNA for translation in plant mitochondria

**Figure 1:**
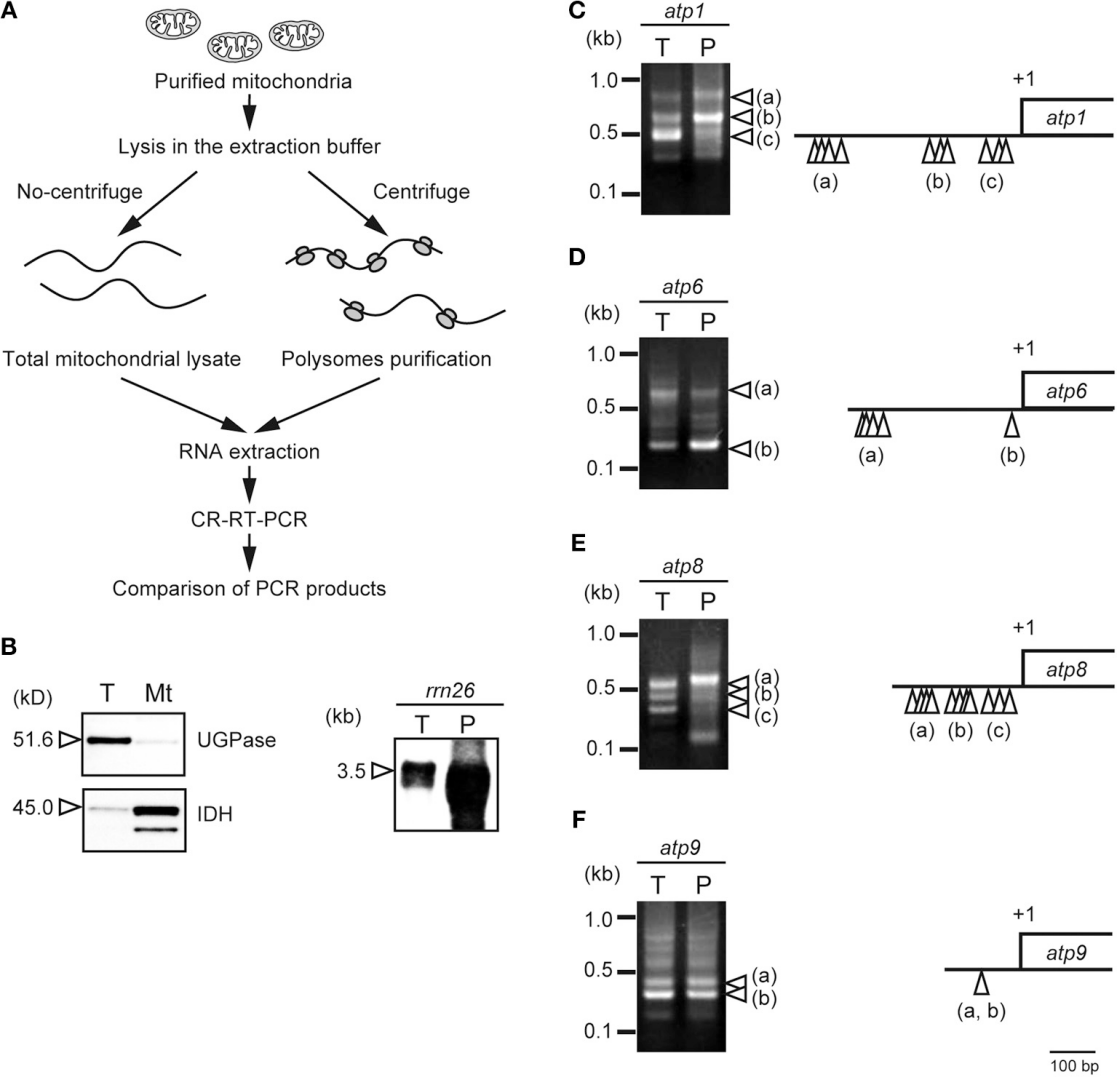
**Heterogeneity in the 5′-end of mRNA and its involvement in translational efficiency in plant mitochondria. (A)** Overview of the CR-RT-PCR analysis compared mitochondrial total RNA with the polysomal RNA. The rice mitochondria were purified as described (Kazama et al., 2008). Purified mitochondria were homogenized in extraction buffer [0.1 M Tris-HCl (pH 7.5), 0.2 M KCl, 35 mM MgCl_2_, 1% triton X-100, 0.5 mg/ml heparin, 3 mM DTT, 0.5 mg/ml chloramphenicol, 25 mM EGTA, 0.2 M sucrose, 2% polyoxyethylene-10-tridecyl ether]. The suspension was centrifuged for 5 min at 20,000 × *g* and 4°C, twice to remove cell debris. The cell debris was further removed by centrifugation for 30 min at 30,000 × *g* and 4°C, twice. The resultant supernatant was transferred to a new tube and centrifuged for 3 hours at 100,000 × *g* and 4°C in TLA100 rotor (Beckman). The precipitate was used for polysomal fraction. **(B)** Purity of the isolated mitochondria was examined by a cytosolic marker protein of (UGPase, UDP-glucose pyrophosphorylase) and a mitochondrial marker protein (IDH, isocitrate dehydrogenase) (left panel). The western blot analyses were performed using total protein (T) and isolated mitochondria (Mt) using the specific antibodies (Agrisera). The enrichment of ribosomes in the polysomal fraction was also assessed by northern blot analysis in total mitochondrial RNA (T) and polysomal mitochondrial RNA (P) using *rrn26* probe (right panel). The images of CR-RT-PCR were shown for mRNAs of *atp1* **(C)**, *atp6* **(D)**, *atp8* **(E)**, and *atp9* **(F)**. The 5′ terminal of each transcript is indicated by white arrowheads in the CR-RT-PCR images mapped on the schematic gene structure (right panel).

Forner et al. (2007) reported that *Arabidopsis* mitochondrial RNAs tend to have heterogeneous 5′-ends but uniform 3′-ends. We conducted an analysis using circularized (CR) reverse transcriptase (RT) PCR to determine whether the heterogeneity of the 5′-end of mRNA is involved in translational efficiency. We compared the 5′-ends of mRNA derived from total mitochondrial RNA with those of mRNA during translation. The RNAs during translation (i.e., the RNAs associated with polysomes) were fractionated by centrifuging at 100,000 × *g* (Figure 1A). The purity of the mitochondria and the enrichment of ribosomes in the polysomal fraction used this study were verified by the western and northern blot analyses (Figure 1B). Next, the RNA termini were determined by CR-RT-PCR for several genes in rice mitochondria (*atp1*, *atp6*, *atp8*, and *atp9*; Figure 1).

The CR-RT-PCR analysis revealed the three major signals for *atp1* (Figure 1C; a, b, and c). Sequence analysis revealed that the 3′-ends are uniform (+141/+142 relative to the stop codon). The multiple 5′-ends were mapped at −390 to −453 relative to the start codon (Figure 1C; a), −167 to −207 (Figure 1C; b), and −34 to −78 (Figure 1C; c). Interestingly, the CR-RT-PCR profile was different in the polysome-associated RNA. The *atp1*-b RNA was most enriched in the polysomal fraction, whereas the *atp1*-c RNA was predominant in the total mitochondrial RNA, suggesting that the heterogenity of the 5′-end could be involved in the translational efficiency of plant mitochondrial RNA.

Similar observations have been made in other mRNAs. The *atp6* RNA accumulated in two forms (Figure 1D; a and b). Their 5′-ends were mapped at around −300 for *atp6*-a and –24/–23 for *atp6*-b, relative to the start codon. The 3′-ends of all cDNAs for *atp6* have been mapped in the dense region (+27 to +29, relative to the stop codon). Polysomal analysis revealed that the shorter form of RNA (*atp6*-b) seems to be more enriched in polysome than the longer one, as shown by the distinct CR-RT-PCR profiles of total RNA vs. polysomal RNA. The *atp8* RNA was shown to accumulate in three different forms with the 5′-ends at −240 to −199, −171 to −113, and −76 to −19 (Figure 1E; a, b, and c, respectively). The 3′-ends were mapped at the same position in all the RNA species (+119/+121, relative to the stop codon). The longest RNA (Figure 1E; a) was concentrated in the polysomal fraction. The *atp9* contains two major isoforms with identical 5′ termini (−85/−84, relative to the start codon) but with different 3′ termini (+110/+111 and +6/+9) (Figure 1F; a and b, respectively). Polysome analysis suggested that the different 3′-end status is not involved in translation.

Together, these results suggested that the heterogeneity of 5′-ends could be involved in translational efficiency in plant mitochondria. Preliminary *in silico* searches have failed to find conserved motifs within the putative translational active RNAs.

## Mitochondrial translation in yeasts and humans

The status of the 5′ UTR differs among different species. As in plants, the mitochondrial mRNAs of the yeast *Saccharomyces cerevisiae* possess characteristic 5′ and 3′ UTRs. The *S. cerevisiae* would be regarded as the best system studying mitochondrial translation. Currently, tens of the translational activators have been identified for the several mitochondrial transcripts (Herrmann et al., 2013). For instance, PET309, which is a membrane-bound PPR protein, acts on the 5′ UTR of the *cox1* mRNA to activate translation and is required to stabilize the precursor of *cox1* RNAs (Manthey and McEwen, 1995). A series of mutations in PPR motifs within PET309 revealed that the PPR motifs are necessary for *cox1* mRNA translation, but not for stabilization (Tavares-Carreón et al., 2008). Thus, the PPR motifs of PET309 may induce a particular RNA conformation to attract and/or interact with the translational machinery. This evidence indicates that mitochondrial translation in *S. cerevisiae* is mainly controlled by the gene-specific translation activator through its association with the 5′ UTR in mRNA (Gruschke and Ott, 2013; Herbert et al., 2013).

Human mitochondrial genes are transcribed using three promoters. The RNAs are subsequently processed and polyadenylated to generate the stop codons (Rorbach and Minczuk, 2012). Mapping of the 5′-end of human mitochondrial mRNAs revealed that mRNAs start directly at or very near the start codon (Montoya et al., 1981). Thus, their mRNAs lack the ribosome-binding site at the 5′ UTR. The analysis of secondary structure at the 5′-ends indicated that the 5′-ends of all mRNA are highly unstructured (Jones et al., 2008). The mechanism of human mitochondrial translation is poorly understood (Koc and Koc, 2013).

Plant mitochondrial RNAs have long 5′ UTRs with no obvious conserved motifs, suggesting that gene-specific translational regulation occurs, as in *S. cerevisiae*. PPR proteins are believed to play a pivotal role in the translation regulation via the 5′ UTR in plant mitochondria.

## Perspective

Translation is a critical step that determines the final level of protein production. Recent research has suggested that mitochondrial gene expression is important in various plant phenomena, such as the pollen production, stress response, germination, and metabolite synthesis.

It is critical to develop techniques to analyze the mitochondrial translational system. The recently developed “genome editing” technology, which has been used to study the human mitochondrial genome, may be applicable to studies of transformation in plant mitochondria (Bacman et al., 2013). Alternatively, incorporation of exogenous RNA into mitochondria using the import pathway for tRNA or via PNPase may facilitate analysis of the *cis*-regulatory element (Wang et al., 2010; Mahato et al., 2011). The tRNA import pathway is applicable for plant as already shown (Sieber et al., 2011; Val et al., 2011). These approaches will facilitate elucidation of the plant mitochondrial translational system and understanding of the diverse methods of mitochondrial translation among different organisms.
